# Supplementary material for: Development of a computer-aided design software for the quantitative evaluation of aesthetic damage
Source: PLoS One. 2019 Dec 18;14(12):e0226322. doi: 10.1371/journal.pone.0226322 (PMC6919621; doi:10.1371/journal.pone.0226322)
Supplement: S1 Code site — 1. (ZIP) [file pone.0226322.s001.zip › [EESC_jr][Projeto]Relatorio_07_21/reports/templates_zip/gentelella-master/vendors/gauge.js/index.html]

gauge.js


# gauge.js

## 100% native and cool looking animated JavaScript/CoffeeScript gauge

coffee

gauge.coffee
gauge.min.js

## Example

#### Variant selection


#### Options:

Current Val:  
Anim speed:  
  
Angle:  
Line width:  
Ptr length:  
Ptr color:  
Ptr stroke:  
Font size:  
Color start:  
Color stop:  
Background:  
  


**Share it!** If checked, the option values will be stored in the URL so that you can easily share your settings.

## Features

- No images, no external CSS - pure canvas
- No dependencies (jQuery is supported, but not required)
- Highly configurable
- Resolution independent
- Animated guage value changes (!)
- Works in all major browsers
- MIT License

## Usage

```
var opts = {
  lines: 12, // The number of lines to draw
  angle: 7, // The length of each line
  lineWidth: 5, // The line thickness
  pointer: {
    length: 10, // The radius of the inner circle
    strokeWidth: 0, // The rotation offset
    color: '#000000' // Fill color
  },
  limitMax: 'false',   // If true, the pointer will not go past the end of the gauge
  colorStart: '0',   // Colors
  colorStop: '0',    // just experiment with them
  strokeColor: '0',   // to see which ones work best for you
  generateGradient: true
};
var target = document.getElementById('foo'); // your canvas element
var gauge = new Gauge(target).setOptions(opts); // create sexy gauge!
gauge.maxValue = 3000; // set max gauge value
gauge.animationSpeed = 3000; // set animation speed (32 is default value)
gauge.set(1500); // set actual value
```

The `Gauge` class handles drawing on canvas and starts the animation.

## Advanced options

- **Percentage color**

  If you want to control how Gauge behavaes in relation to the displayed value you can use the Guage option called **percentColors**. To use it add following entry to the options:

  ```
  percentColors = [[0.0, "#a9d70b" ], [0.50, "#f9c802"], [1.0, "#ff0000"]];
  ```

  see working example at JSFiddle

### jQuery plugin

Gauge.js does not require jQuery. Anyway, if you use jQuery you may use the following plugin:

```
$.fn.gauge = function(opts) {
  this.each(function() {
    var $this = $(this),
        data = $this.data();

    if (data.gauge) {
      data.gauge.stop();
      delete data.gauge;
    }
    if (opts !== false) {
      data.gauge = new Gauge(this).setOptions(opts);
    }
  });
  return this;
};
```

## Supported browsers

Gauge.js has been (not yet!) successfully tested in the following browsers:

- Chrome
- Safari 3.2+
- Firefox 3.5+
- IE 9
- Opera 10.6+
- Mobile Safari (iOS 3.2+)
- Android 2.3+

## Changes

### Version 1.2.1 (9.03.2014)

- Proper handling of color params issue #47.
- Moved percentage color to example/docs + JSFiddle

### Version 1.2 (16.08.2012)

- Prototype chain fix. See issue #7.
- Refactored code a bit to make it more flexible. Default class that has some extra features like gradient shadows is called Donut while more flexible one (for devs) is called BaseDonut - use it if you would don't need extra automatic stuff.
- Ability to scale gauges (requested via email) - example at JSFiddle

### Version 1.1 (15.08.2012)

- Fixed color picker bug in FF & Opera
- Added a shadow option. See issue #5.
- Added multiple pointer option (requested via email). This needed some code refactoring. No demo for it yet. Use array of values to check it ex. gauge.set([44, 554]);
- Added wrapper for formatting text output issue #4.

### Version 1.0 (27.6.2012)

- Initial release

## Contact

If you encounter any problems, please use the GitHub issue tracker.  
If you like gauge.js and use it in the wild, let me know.  
If you want to contact me, drop me a message via email

Hosted on GitHub
